# Apixaban (Eliquis)-Induced Rash: A Case Report

**DOI:** 10.7759/cureus.50273

**Published:** 2023-12-10

**Authors:** Harshita Nadella, Nicole Vilar, Ross Nochimson

**Affiliations:** 1 Rheumatology and Immunology, Nova Southeastern University Dr. Kiran C. Patel College of Osteopathic Medicine, Davie, USA; 2 Internal Medicine, Nova Southeastern University Dr. Kiran C. Patel College of Osteopathic Medicine, Davie, USA; 3 Dr. Kiran C. Patel College of Osteopathic Medicine, Nova Southeastern University, Fort Lauderdale, USA; 4 Internal Medicine, Internal Medicine Clinic, Lauderhill, USA

**Keywords:** eliquis, adverse effect, embolism, rash, apixaban

## Abstract

Many anticoagulants are indicated as prophylaxis and treatment for conditions that may lead to thromboembolisms or atrial fibrillation. Among those, apixaban, a reversible direct inhibitor of factor Xa, is one of the most popular. Apixaban is known to cause a variety of side effects; however, for this paper, the focus is on hypersensitivity reactions. Although several cases will be referenced from the literature, we present a case with a history of a dermatological disease before the use of a direct oral anticoagulant. A 74-year-old female patient with a history of pemphigus vulgaris and a diagnosis of pulmonary embolism and right distal vein thrombosis managed with apixaban presented with a pruritic coalescent erythematous dermatitis throughout her torso, back, and lower extremities three days post-apixaban treatment. In accordance with her physical examination, the apixaban regimen was withdrawn, and oral dabigatran, a direct thrombin inhibitor, was initiated. At the patient’s five-day follow-up, clinical improvement was noted. Through clinical diagnosis, it is believed that this dermatitis was a reaction to the apixaban treatment course.

## Introduction

Apixaban, a reversible direct inhibitor of factor Xa, is an anticoagulant indicated as prophylaxis and treatment for thromboembolisms and non-valvular atrial fibrillation. Apixaban has several clinical side effects encompassing major and non-major bleeding, vestibular symptomatology, and hypersensitivity rashes [1]. Although several cases of hypersensitivity reactions secondary to apixaban use have been reported, we present a case of possible apixaban-induced hypersensitivity reaction categorized as drug-induced eruptive dermatitis.

## Case presentation

A 74-year-old Caucasian female patient with a history of pemphigus vulgaris managed with prednisone and mycophenolate presented to the emergency room with a fever of 100.8°F, cough, and fatigue. She was diagnosed with viral pneumonia, an upper respiratory infection, and COVID-19. Respiratory panel results, lab values, and viral titers were currently unknown. Per the discharge notes provided by the patient, it was believed that the viral pneumonia diagnosis was secondary to the COVID-19 diagnosis. On discharge, she was prescribed 6 mg of dexamethasone for seven days and 200 mg of benzonatate three times per day. No other antiviral treatment was provided at this time. Four days after discharge, she presented to her primary care physician with bilateral lower leg edema and shortness of breath, for which she was advised to return to the emergency room. In returning to the emergency room, a D-dimer test and venous ultrasound were performed to diagnose a deep vein thrombosis (DVT). The patient’s D-dimer was elevated, and the venous ultrasound of her right lower extremity was positive for a DVT. Due to the patient’s shortness of breath, further evaluation to rule out a pulmonary embolism (PE) was performed. An arterial blood gas (ABG) and chest X-ray (CXR) were initially performed, and respiratory alkalosis was seen along with an area of right lung parenchyma lucency. A CT pulmonary angiography was performed which confirmed the diagnosis of a small right PE. The patient was then discharged on a five-week regimen of apixaban (10 mg twice/day for seven days, followed by 5 mg oral tablets every 12 hours). On day three of her regimen, she presented to a walk-in urgent care clinic with a pruritic erythematous full-body rash and a continuation of shortness of breath. Upon physical examination, she was noted to have a well-circumcised serpiginous and coalescent erythematous rash that encompassed the entirety of her torso and back while her lower extremities showed multiple erythematous plaques distributed bilaterally on the anterior portion of her shins suggestive of eruptive dermatitis.

Diagnostic studies

At the time of presentation, diagnostic studies such as immunoglobulin E (IgE) levels, a complete blood count (CBC), or biopsies, were deemed unnecessary. Of note, the patient stated that she autonomously discontinued her prednisone and mycophenolate three months before this episode. She was being monitored by her rheumatologist for her pemphigus vulgaris. Therefore, through oral confirmation, a flare-up of pemphigus vulgaris was eliminated from the differential as the patient confirmed that her disease typically presented with the classical flaccid intradermal blisters, which were not present during physical evaluation. Regrettably, at this time we did not have access to previous notes, labs, or procedures performed by her previous physicians due to physician office closures or state-to-state transfer of information. Therefore, the patient’s past medical history was limited to the most recent rheumatology note which stated her diagnosis of pemphigus vulgaris and an oral dictation provided by the patient. It is important to note that the patient confirmed she had no prior history of use of any anticoagulation treatment.

Treatment

In accordance with her physical examination, the apixaban regimen was withdrawn, and oral dabigatran, a direct thrombin inhibitor, was initiated. Additionally, 0.05% clobetasol propionate ointment, a topical corticosteroid, was incorporated because of suboptimal results.

Outcome

At the patient’s five-day follow-up, clinical improvement was noted. As previously mentioned, no diagnostic studies were performed before the regimen change, nor were they performed during treatment. Based on this improvement, they were deemed unnecessary. The patient was maintained on this regimen until she was asymptomatic and as prophylaxis for PE and DVT per the American Society of Hematology. Hence, per the patient’s clinical symptomatology and past medical history, a clinical diagnosis of drug-induced eruptive dermatitis was made.

## Discussion

Apixaban, a reversible direct inhibitor of factor Xa, is an anticoagulant indicated as prophylaxis and treatment for thromboembolisms and non-valvular atrial fibrillation. It is one of the most commonly used anticoagulants in populations with the aforementioned conditions. Apixaban has several clinical side effects encompassing major and non-major bleeding, vestibular symptomatology, and hypersensitivity rashes - the key factor in this paper. To distinguish the causative agent for this rash, it is imperative to take a closer look at the various presentations of rashes induced by apixaban.

Many drugs are known to have cutaneous eruptions as common adverse effects; up to 2-3% of active drugs on the market have associated drug reactions as side effects. Cardiovascular drugs such as beta-blockers, angiotensin-converting enzymes, and digoxin are high on this list but anticoagulant drugs are rarely associated. Factor Xa plays a crucial role in the coagulation cascade as it is part of the common pathway, a converging point of the intrinsic and extrinsic pathways that ultimately lead to form thrombin and fibrin. As apixaban blocks factor Xa, there is a decrease in epidermal cyclic adenosine monophosphate levels which correlates to a favorable psoriasiform drug eruption [1]. Furthermore, this causes a downstream effect of delayed hypersensitivity and impaired lymphocyte transformation prolonging the rash’s effects and durations [2]. Additionally, it is vital to note and recognize this patient’s pre-existing history of pemphigus vulgaris which could exacerbate the mechanisms discussed above.

There have been other circumstances in which a rash resulted after the use of apixaban. In a 2017 study to evaluate the safety of apixaban as thromboprophylaxis in bariatric surgery, 1.7% of patients experienced apixaban-related side effects. The most common side effects noted in this population were menorrhagia and a rash [3]. This study concluded that apixaban was a safe choice due to minimal side effects and prevention of thromboembolic events. Similarly, in 2020, a woman was noted to have developed “small areas of red rash that appeared bilaterally on her lower extremities” upon being placed on apixaban for DVT treatment [4]. This rash presented 23 days after being on the treatment and promptly resolved after her medication was switched to rivaroxaban, a direct factor Xa inhibitor. Even though dermatologists referred to this rash as an apixaban-induced leukocytoclastic vasculitis, the etiologies between this case and the case being presented share vast similarities. In contrast, a case in 2017 documented a female patient who had been placed on a prophylaxis rivaroxaban regimen for atrial fibrillation and developed a hypersensitivity reaction to it [5]. These symptoms resolved when she was switched from rivaroxaban to apixaban. As the patient had no prior history of an allergic reaction to rivaroxaban or a documented history of a similar reaction while on similar medications, it was concluded that the mechanism of this rash was unclear, specifically because the patient was switched to medication within the same class. Although resolution was successful in these patients, several studies have demonstrated that switching between direct factor Xa inhibitors may not be the appropriate alternative for all patients.

Anis et al. and El-Sabbagh et al. demonstrated two separate cases of patients with past medical histories of hypercoagulability leading to the chronic use of anticoagulation therapy that both presented with a rash when switched from their prior anticoagulation treatment to a direct factor Xa inhibitor. The patient in the study by Anis et al. initially presented with a rash after being switched from warfarin to apixaban. The patient was then switched from apixaban to rivaroxaban, which exacerbated his rash. Upon reversal to his warfarin therapy, the rash resolved over time. Similarly, the patient in the study by El-Sabbagh et al. presented when he was switched from rivaroxaban to apixaban, and had a resolution when he was discontinued from apixaban and initiated on warfarin. Both of these cases led to the possibility that there may be a cross-reactivity between apixaban and rivaroxaban leading to the worsening of a hypersensitivity reaction from these medications [6,7]. The differential diagnoses discussed above are summarized in Table 1 [8].

**Table 1:** The differential diagnoses for the apixaban-induced rash.

Differential diagnoses	Pathophysiology
Drug-induced eruptive dermatitis due to apixaban	Hypersensitivity reaction due to decreased epidermal cyclic adenosine monophosphate
Apixaban-induced leukocytoclastic vasculitis due to apixaban	Delayed hypersensitivity dermatological reaction due to further downstream signaling reaction leading to impaired lymphocyte transformation
Hypersensitivity reaction due to rivaroxaban	Hypersensitivity reaction due to decreased epidermal cyclic adenosine monophosphate
Cross-reactivity between apixaban and rivaroxaban	Cross-reactivity between the mechanisms of both drugs leads to a hypersensitive reaction
Allergic reaction (type 1) to apixaban	Immunological release of chemical mediators from mast cells and basophils after prior sensitization to an allergen
Type 4 hypersensitivity to apixaban	T-antigen interaction that causes activation and cytokine secretion that respond 24–48 hours after exposure to soluble antigen

Therefore, although first-line for the prophylaxis and treatment of patients with thromboembolisms and a history of atrial fibrillation, direct reversible factor Xa inhibitors, such as apixaban and rivaroxaban, should be prescribed with the knowledge that a cutaneous eruption may present secondary to a hypersensitivity reaction and that switching among the class may exacerbate the initial reaction. Physicians should weigh the benefits of these DOACs in accordance with the patient’s past medical history and determine whether a direct thrombin inhibitor instead of a direct factor Xa inhibitor may provide the same benefit without the cutaneous side effect. Should a physician deem the benefits outweigh the risks of a direct factor Xa inhibitor, the patient should be warned of the possibility of a cutaneous eruption and reassured that a change in DOAC choice may resolve the eruption.

## Conclusions

A Caucasian female patient developed drug-induced dermatitis while taking apixaban for the treatment of a PE/DVT. There are many possible immunological explanations for drug-induced rash (specifically apixaban), as discussed above; however, this patient was deemed to have drug-induced eruptive dermatitis. This was done largely through prompt clinical reasoning. After the apixaban regimen was withdrawn and the oral dabigatran was started, her rash reduced and her symptoms were relieved. Through exploration of various clinical studies, it was found that apixaban causes this delayed hypersensitivity dermatological reaction due to decreased epidermal cyclic adenosine monophosphate and further downstream signaling reaction leading to impaired lymphocyte transformation. As a result, physicians must look at a patient’s history for signs of hypersensitivity and weigh the risks and benefits of prescribing apixaban before the patient exhibits symptoms. It will also be beneficial to further explore a more detailed reasoning behind this dermatitis to exhibit any conditions that may increase the chances of exhibiting this rash.
